# Comparative Anatomy of the Mus Musculus

**Published:** 1831

**Authors:** 


### LXXIII.

### Comparative Anatomy of the Mus Musculus. By H. W. Dewhurst, Esq. 12mo, pp. 14.

This little brochure is very appropriately inscribed to His Royal Highness the Duke of Sussex, who presides over a society, where, if fame speak true, there is a large stock of—

"Rats and Mice, and such small deer,"

that may afford the Royal Patron ample field for *comparative* anatomy, in the department to which Mr. Dewhurst has directed his attention. Mr. D. has given

---

a minute and scientific anatomy of a little animal whose external form, nimble march, and furtive propensities, are but *too well* known to man. The author considers it "rather surprising that, in the present state of science, and march of intellect, so little attention should be paid to the anatomy of the various animals by which we are surrounded." But when we reflect on the propensity which man, in all ages, has evinced for dissecting his fellow-creature, we need hardly be surprised to find rats, mice, and the inferior animals left to the care of professed *catch-ers* of a description somewhat different from those who are technically termed "BODY-SNATCHERS." We wish Mr. Dewhurst success in his undertakings.

---

### LXXIV.

Tracheotomy, and permanent breathing through a Tube. By Mr. J. Kenworthy.

